# Detection of human antibodies binding with smooth and rough LPSs from *Proteus mirabilis* O3 strains S1959, R110, R45

**DOI:** 10.1007/s10482-017-0937-0

**Published:** 2017-09-09

**Authors:** J. Gleńska-Olender, K. Durlik, I. Konieczna, P. Kowalska, J. Gawęda, W. Kaca

**Affiliations:** 1 Institute of Biology, Jan Kochanowski University, 25-406, Kielce, Poland; 2 Świętokrzyskie Biobank, The Regional Science and Technology Center, 26-060 Podzamcze, Poland; 3 Świętokrzyskie Rheumatology Centre, St. Lukes Hospital, 26-200, Końskie, Poland

**Keywords:** *Proteus mirabilis*, LPS, Rheumatoid arthritis Abs

## Abstract

Bacteria of the genus *Proteus* of the family *Enterobacteriaceae* are facultative human pathogens responsible mainly for urinary tract and wound infections, bacteremia and the development of rheumatoid arthritis (RA). We have analyzed and compared by ELISA the titer of antibodies in plasmas of healthy individuals and in sera of rheumatoid arthritis patients recognizing a potential host cross-reactive epitope (lysine-galacturonic acid epitopes) present in *Proteus* lipopolysaccharide (LPS). In our experiments LPSs isolated from two mutants of smooth *Proteus mirabilis* 1959 (O3), i.e. strains R110 and R45, were used. R110 (Ra type mutant) is lacking the O-specific polysaccharide, but possesses a complete core oligosaccharide, while R45 (Re type) has a reduced core oligosaccharide and contains two 3-deoxy-d-*manno*-oct-2-ulosonic acid residues and one of 4-amino-4-deoxy-l-arabinopyranose residues. Titer of *P. mirabilis* S1959 LPS-specific-antibodies increased with the age of blood donors. RA and blood donors’ sera contained antibodies against S and Ra and Re type of *P. mirabilis* O3 LPSs. Antibodies recognizing lysine-galacturonic acid epitopes of O3 LPS were detected by ELISA in some plasmas of healthy individuals and sera of rheumatoid arthritis patients. RA patients antibodies reacting with *P. mirabilis* S1959 S and R LPSs may indicate a potential role of anti-LPS antibodies in molecular mimicry in RA diseases.

## Introduction

*Proteus mirabilis* strains belong to the family *Enterobacteriaceae*. It is a Gram-negative bacillus, a facultative anaerobe found in soil, polluted water as well as a part of the normal microflora of human and animal intestines. *P. mirabilis* strains are responsible for urinary tract infections and can lead to rheumatoid arthritis (RA) (Rózalski et al. 1997; Rózalski et al. 2012; Wilson et al. 1995). Human antibodies (Abs) act by recognizing bacterial pathogens preventing the spread of diseases. However, due to molecular mimicry of bacterial and human epitopes anti-bacterial Abs might be responsible for auto-immune reactions that can led to deterioration of patient’s tissues (Ebringer et al. 2010; Wilson et al. 1995), observed with anti-bacterial urease and hemolysin Abs (Ebringer et al. 2010; Konieczna et al. 2012; Rashid and Ebringer 2007). The virulence factors of *P. mirabilis* include lipopolysaccharides (LPSs) (Rózalski et al. 1997). Depending on the structure of the O-polysaccharide part of LPSs, *Proteus* spp. strains are currently classified into 80 different O-serogroups (Knirel et al. 2011; Siwinska et al. 2015). The peculiar features of *Proteus* O-antigens are non-carbohydrate substituents, such as amino acids and their derivatives (Knirel et al. 2011). One of such non-carbohydrate epitopes is l-lysine linked to the carboxyl group of d-galacturonic acid [6*N*-α-(d-galacturonoyl)-l-lysine, Lys-GalA] in *P. mirabilis* O3 LPS (Kaca et al. 1987; Gromska and Mayer 1976). Previous studies have shown the significance of an Lys-GalA amide for serological specificity of O-antigens and the core regions of *P. mirabilis* LPS. This epitope plays a crucial role in serological cross-reactions of *P. mirabilis* 028, S1959 LPSs and *P. mirabilis* R14/S1959 with heterologous polysaccharide-specific antisera (Palusiak et al. 2014; Radziejewska-Lebrecht et al. 1995). The above mentioned studies utilized rabbit anti-LPSs Abs. The prevalence of anti-Lys-GalA Abs in human sera has not been studied.

In our previous studies we have shown that the amount of anti-S1959, -R110 and -R45 LPSs Abs in RA patients in 1: 1000 diluted sera were higher than in control healthy donors sera (Arabski et al. 2012). The focus of the present studies was to find out if human Abs recognize the Lys-GalA epitope present in the O-polysaccharide of LPS of *P. mirabilis* O3 strain S1959 as well as serological epitopes present in the core and lipid A parts of O3 LPS. Smooth O3 LPS and its two Ra and Re type mutants (R110 and R45, respectively), missing Lys-GalA residues, were chosen for the present study, in which Abs titers against *P. mirabilis* O3, its two R mutants and synthetic Lys-GalA were established.

## Materials and methods

### Human plasma and sera

Plasma samples were taken from 102 healthy donors (VBD), 28 women and 74 man, with mean ages 33 ± 10.6 years (Regional Blood Center Kielce, Poland). Serum samples were taken from 14 healthy donors aged 33 ± 9.1 (Regional Blood Center, Kielce, Poland) and 14 patients with rheumatoid arthritis at age 56.7 ± 14.3 (hospitalized in the Świętokrzyskie Rheumatology Centre, St. Lukes Hospital, Końskie, Poland). Two selected sera of patients (strong ELISA reaction with LPS S1959) were purified to the IgG Ab fraction (Protein A IgG Purification Kit, Thermo Scientific).

### LPS antigens

LPSs from three *P. mirabilis* strains were used at a concentration of 0.5 mg/mL in phosphate buffered saline (PBS), pH 7.4: S1959 (wild type; Kaca et al. 1987), R110 (Ra mutant of S1959, no O-antigen; Radziejewska-Lebrecht and Mayer 1989; Vinogradov et al. 2000), and R45 (Re mutant having only a fragment of the inner core; Sidorczyk et al. 1983). All LPS were purified as reported earlier (Żarnowiec et al. 2017).

### Synthetic antigen

The synthetic 2-acrylamidoethyl α-glycoside of Lys-GalA, representing a partial structure of the *P. mirabilis* O3 O-antigen, was co-polymerized with acrylamide (PAA) (Chernyak et al. 1994).

### Immuno-enzymatic test

The titers of anti-LPS *P. mirabilis* Abs were determined in healthy donors and patient plasma and sera by ELISA. Microtiter flat bottom plates (Pierce; NUNC; Thermo Fisher Scientific) were coated with LPS (0.5 µg per well), diluted in PBS (pH 7.2) and left at 4 °C overnight. The plates were washed with PBS, blocked with 2% BSA (Sigma, USA) at 37 °C for 2 h and then again washed three times with PBS. The plates were incubated with human sera/plasma diluted from 1/200 to 1/125600 in PBS with 1% BSA for 1 h at 37 °C followed by washing three times with PBS. Samples were tested in triplicate. After incubation at 37 °C for 1 h and washing, the second Abs [either goat anti-human IgG or IgM, conjugated with peroxidase (Sigma, USA), diluted 1:1000 in PBS with 1% BSA] were added. Subsequently, plates were washed three times with PBS. In the final stage, O-phenylenediamine dihydrochloride FAST (Sigma, USA) was added which is a substrate for peroxidase. After incubation for 20 min at room temperature, the reaction was stopped by addition of water solution of 0.5 M H_2_SO_4_ acid and the absorbance was measured at 492 nm by a multi-mode microplate reader (Infinity, Tecan).

As controls in ELISA, the nonspecific reactions plasma/serum (in proper dilution) + II Ab without antigen were performed and the absorbance for each serum or plasma dilution was subtracted. The same procedure was used for isolated human IgG Abs.

### Abs absorption test

Dilutions of 1/1600 (50 µl) of purified IgG fractions from rheumatoid arthritis patient sera 005 and 008 were added to the wells of a polystyrene plate coated with antigen (human collagen type I; Corning, USA). The IgG fractions were applied in six repeats. After 1 h incubation at 37 °C with mixing, 50 µl of IgG fractions were transferred to plates pre-coated with second antigens (S1959 LPS, R110/S1959 LPS or Lys-GalA-PAA) and ELISA were performed. As control plates with human collagen type I, *P. mirabilis* LPSs or Lys-GalA-PAA alone were used.

## Results

### Detection in human plasma of Abs recognizing Proteus mirabilis S1959 (O3), R110 R45 LPSs and Lys-Gal PAA synthetic antigen

**Fig. 1:**
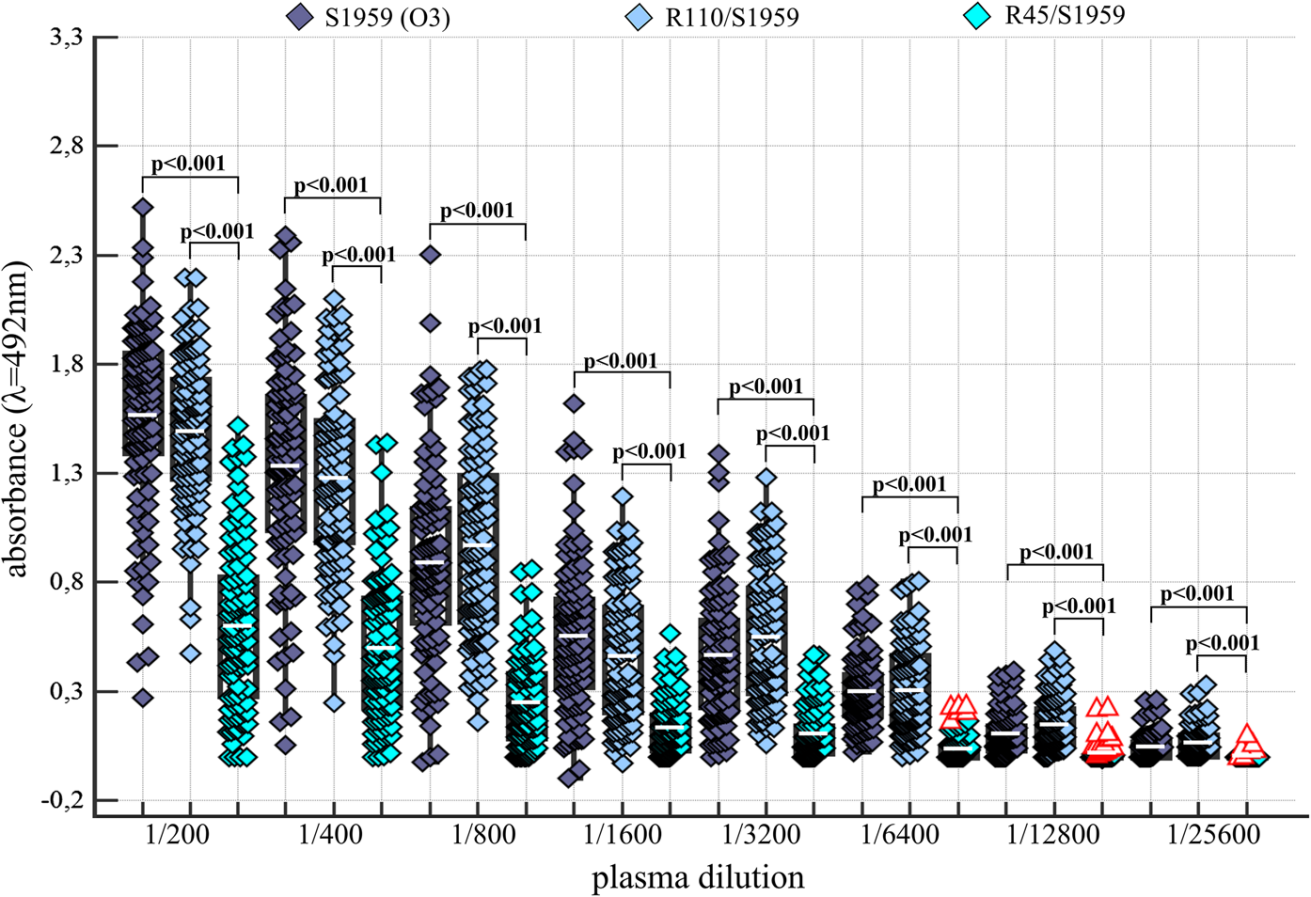
Amount of Abs from serially diluted human plasmas samples reacting with *P. mirabilis* S1959, R110 (Ra) and R45 (Re) LPSs. *Red triangle*, data out of presented range

**Fig. 2:**
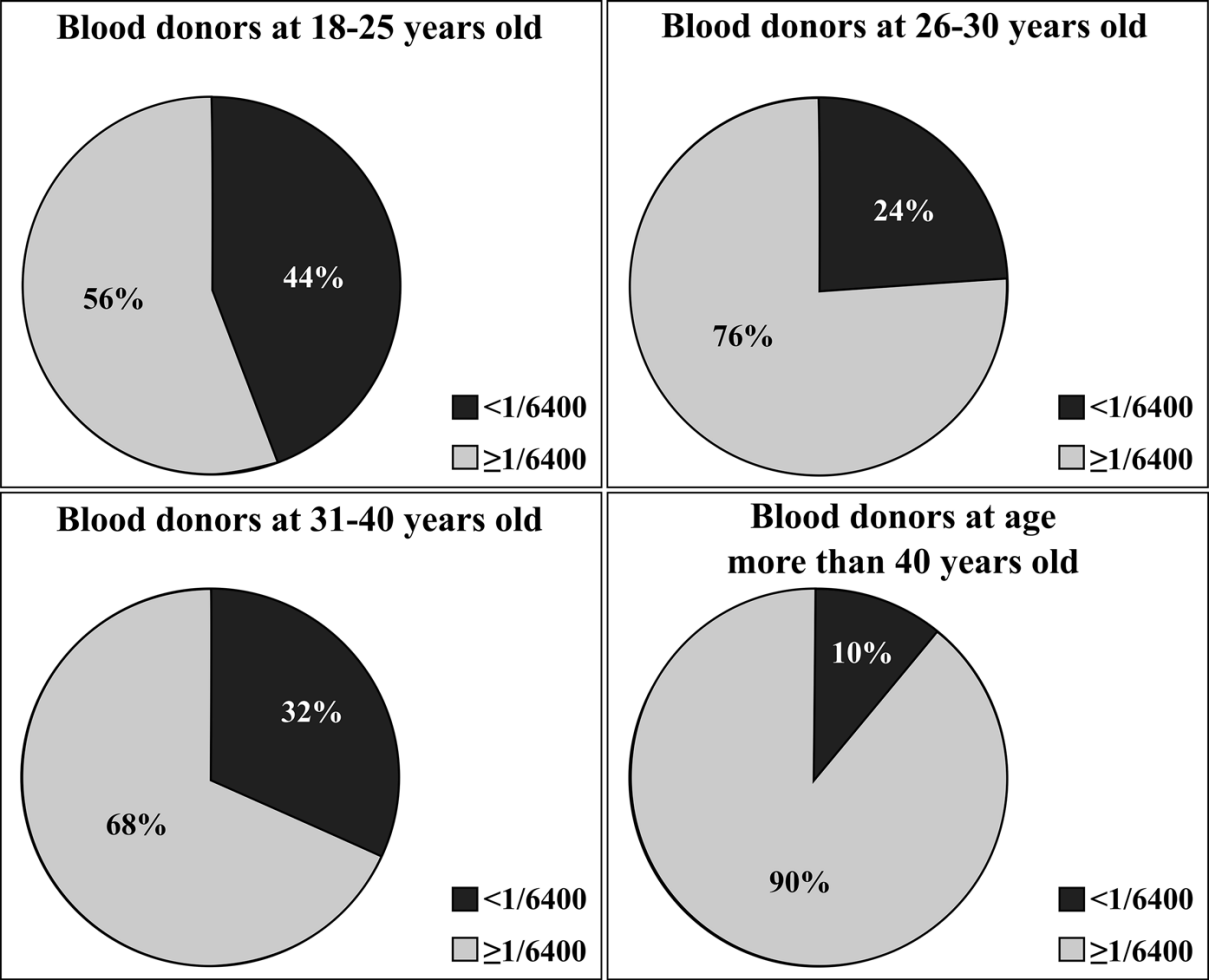
Ab titers against *P. mirabilis* S1959 LPS correlated with the age of plasma donors. **a** 18–25, **b** 26–30, **c** 31–40, **d** more than 40 years old

In order to determine the titer of Abs against LPSs of *P. mirabilis* S1959 and its two mutants R45 (Re) and R110 (Ra), plasma samples obtained from 102 blood donors were examined by ELISA. The results showed that in all tested plasma there were present Abs binding to S1959, R110 (Ra) and R45 (Re) LPSs (Fig. 1). The titer of Abs against S1959 and R110 LPSs were similar up to the dilution 1/6400. For only 1% of plasma the Abs specific to S1959 LPS were detected in dilution 1/800, for 5.8% of plasma the Abs were found in 1/25600 dilution and more than 50% of the plasma samples showed Abs titer 1/6400. Only plasma Abs against Re type of LPS were detected in much lower amount in each dilution, in comparison to S and Ra LPSs (Fig. 1). Moreover, the correlation between the age of donors and Abs level in plasmas against *P. mirabilis* S1959 LPS could be verified (Fig. 2). Results showed correlation between the age of blood donors and Abs level in plasmas against *P. mirabilis* S1959 LPS. The level of Abs rose with age, namely 55% of plasma samples of VBD of 25 years tested with LPS S1959 possessed a level higher or equal to 1/6400 titer while in people over the age of 26, between 68 and 90% were identified (Fig. 2).

In order to obtain a more detailed map of human Abs recognizing the Lys-GalA epitope synthetic antigen was used. The most reactive four plasmas were chosen, based on their ELISA reactivities with *P. mirabilis* LPSs. Results suggested that the Lys-GalA amide coupled with polyacrylamide was bound by Abs in all four tested plasmas in dilution from 1/1600 to 1/6400 (Table 1). This confirmed that this non-carbohydrate epitope is recognized by human Abs present in plasmas.

**Table 1:** Titers of Abs in human plasmas reacting with *P. mirabilis* LPSs and Lys-GalA-PAA synthetic antigen

	Plasma 007				Plasma 065			
Antigen	LPS S1959	Lys-GalA-PAA	LPS R110	LPS R45	LPS S1959	Lys-GalA-PAA	LPS R110	LPS R45
Titer	1/12800	1/1600	1/12800	1/1600	1/25600	1/3200	1/12800	1/3200

	Plasma 090				Plasma 096			
Antigen	LPS S1959	Lys-GalA-PAA	LPS R110	LPS R45	LPS S1959	Lys-GalA-PAA	LPS R110	LPS R45
Titer	1/6400	1/6400	1/12800	1/800	1/6400	1/1600	1/6400	1/800

### The presence of Abs in sera from RA patients binding S and R P. mirabilis LPSs

**Fig. 3:**
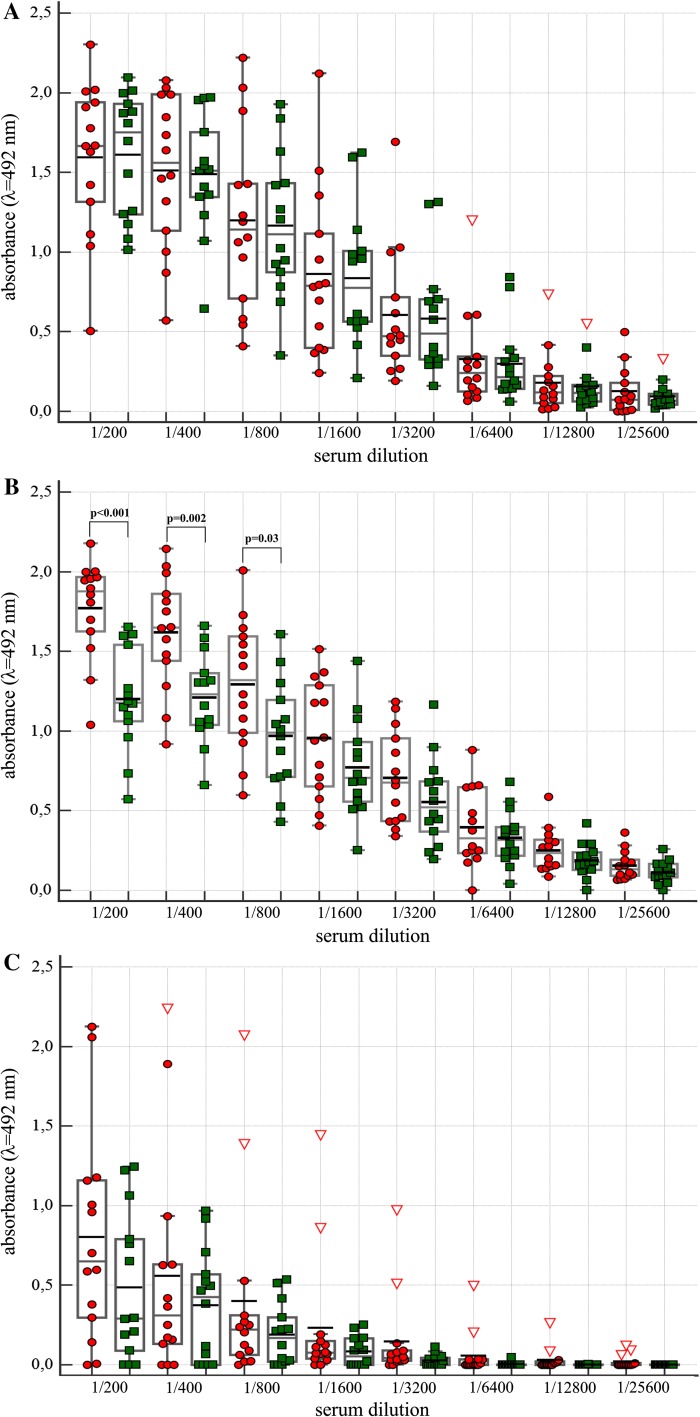
Amount of Abs from serially diluted sera from RA patients and blood donors binding to LPSs from *P. mirabilis* S1959 (**a**), R110 (**b**) and to Lys-GalA-PAA synthetic antigen (**c**)*. Red circles*, *green squares* RA and VBD sera, respectively. *Red triangle*, data out of presented range

**Fig. 4:**
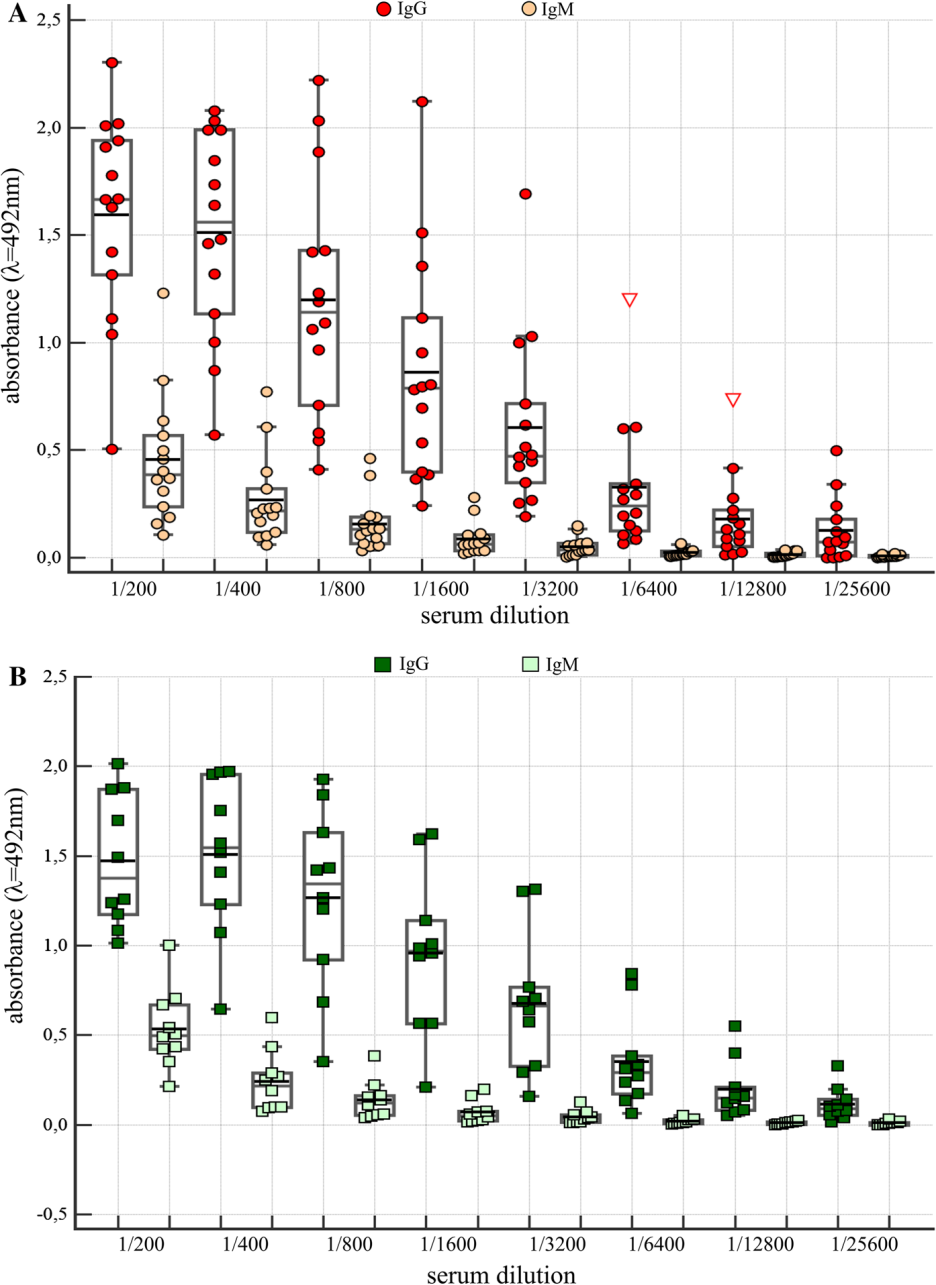
IgG and IgM Abs from sera of RA (*red circles*) patients and VBD (*green squares*) in reaction with *P. mirabilis* S1959 LPS. *Red triangle*, data out of presented range

Since anti-*P. mirabilis* S and R LPSs, and anti-Lys-GalA Abs were common in human plasma samples, in the following experiments sera from 14 RA patients were tested with three *P. mirabilis* O3 antigens. As controls 14 sera of VBD were used. In patients as well as in controls sera Abs against two tested LPSs and Lys-Gal-PAA were present (Fig. 3a–c). The titer (1/800) of Abs that bind Lys-Gal-PAA was much lower than those of anti-R110 and S 1959 LPSs Abs, i.e. 1/6400 (Fig. 3). Only in the case of RA patient sera, Abs reacting with Ra LPS (R110) quantitatively dominated over Abs in VBD sera in the first three dilutions (Fig. 3b). The results presented in Fig. 4a, b indicate that both IgM and IgG Abs in anti-Ra and -VBD sera reacted with S1959 LPS. However, the observed amount of IgM Abs was definitively lower than that of the IgG class (Fig. 4).

**Fig. 5:**
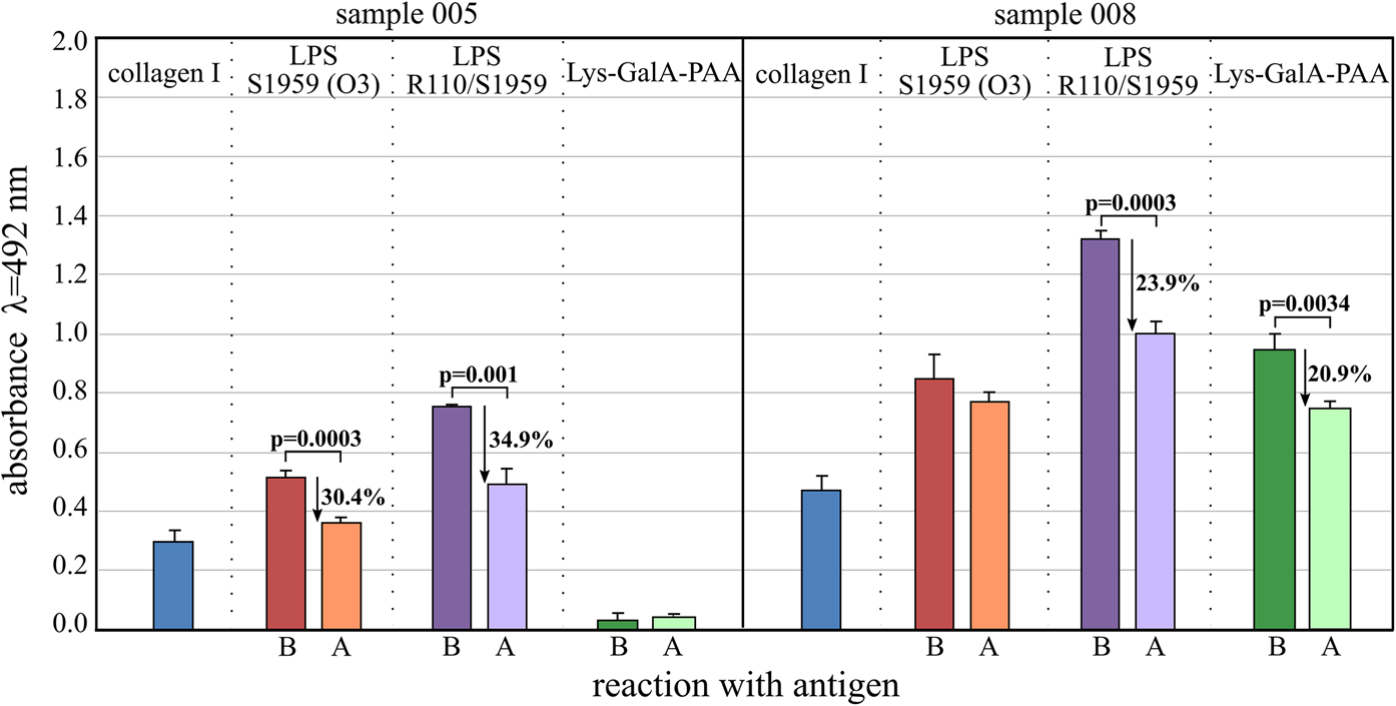
ELISA data for isolated IgG Abs from sera of two RA patients reacting with human collagen type I, LPS of *P. mirabilis* S1959 and R110, and Lys-GalA-PAA synthetic antigen. *Blue bars*, collagen type I alone as control. *B*, reaction before absorption with collagen. *A*, reaction after absorption with collagen

To make the serological reactions more decisive IgG Abs were isolated from the two most reactive patients sera, namely 005 and 008, and tested against synthetic Lys-GalA PAA and *P. mirabilis* O3 LPS antigens (Fig. 5). Human collagen type I was added, comprising exposed lysine residues in positions 337 (Doi et al. 1993) as components of molecular mimicry. We asked the question whether the same Abs from RA patients that bind collagen are reacting with LPSs and Lys-GalA epitopes. To answer this, adsorption experiments were performed. Pre-adsorption with collagen type I significantly reduced binding of at least one of the LPS (Fig. 5). Only in 008 serum Abs against Lys-GalA PAA were detected, however, pre-incubation with collagen diminished reactivities at a rate of 20.1%. In conclusion, IgG Abs bind collagen, *P. mirabilis* S, R LPSs and Lys-GalA PAA antigens.

## Discussion

*P. mirabilis* strains are typical bacteria of the human intestinal microflora, but they may become opportunistic pathogens of urinary tract infections (UTI) under certain conditions. In our previous studies we have shown that Abs against smooth and R type LPS of *P. mirabilis* are frequent in RA and blood donor sera (Arabski et al. 2012). The current ELISA data allowed us to establish anti-*P. mirabilis* LPSs Abs titer in human plasmas as 1/6400 with statistically significant differences between S and R LPSs (Fig. 1). Titers of *P. mirabilis* S1950 LPS-specific-Abs in blood donor plasma increased with their age (Fig. 2) which might be associated with a long exposure to the antigens tested in this study. Earlier, we could show that serotype O3 was one of most frequent clinical *P. mirabilis* isolates from Polish patients (Kaca et al. 2011).

In plasmas as well as in sera from RA patients and blood donors immunochemical reactions are scattered. Significantly more anti-R110 LPS Abs were identified in sera from RA patients than in those from blood donors (Fig. 3b). This is contradictory to previous results showing that the summary level of anti-*P. mirabilis* R110-LPS Abs of RA patients compared to healthy donors were not different (Arabski et al. 2012). Discrepancies might be due to sources of RA sera, i.e. hospital and out-hospital patients, and the number of samples.

The absorption experiment with IgG Abs from the sera of two RA patients, in which collagen type I was used, indicated that only one serum contained Abs against Lys-GalA-PAA. Absorption with collagen type I reduced reactions with LPSs only between 24 and 35%. Our data do not allow us to postulate that Lys-GalA epitopes are involved in RA progression.

One may speculate that sera from RA patients represented a “puzzle of Abs” with varied amount and specificity. The final outcome of the observed ELISA reactions might depend on specific as well as less specific “sticky” Abs (Güven et al. 2014). In addition, ELISA with R and S LPSs depend on the amount of antigen fixed to microplate. We have shown by LAL test that *P. mirabilis* RA LPS binds about 10 times less to a microplate then smooth S1959 LPS (Arabski et al. 2012).

It is suggested by us and others that cross-reactivity between bacterial and human antigens is due to molecular mimicry mechanism, that later on may be involved in RA etiopathogenesis (Lorenz et al. 2013; Christopoulos et al. 2017; Konieczna et al. 2012; Rashid and Ebringer 2007). Abs against *P. mirabilis* were recorded at a significantly high level in sera from RA patients which could be due to frequent UTI and asymptomatic bacteuria (Rashid and Ebringer 2007). Elevated levels of IgM, IgG and IgA Abs were detected in sera from RA patients reacting with *P. mirabilis* UreC, UreF, HpmA, HLA-DR1/4 and collagen type XI peptide epitopes (Christopoulos et al. 2017; Konieczna et al. 2012; Rashid and Ebringer 2007). The presence of anti-*P. mirabilis* LPSs-Abs in sera from RA patients was not subject of previous studies (Rashid and Ebringer 2007). Anti-LPS Abs might be considered as an additional factor of molecular mimicry in RA diseases. More data are needed to determine RA specificity of Abs that react with carbohydrate and non-carbohydrate substituents of *P. mirabilis* LPS.
